# 高效液相色谱-串联质谱法测定电子烟油中咖啡因和牛磺酸

**DOI:** 10.3724/SP.J.1123.2025.09003

**Published:** 2025-12-08

**Authors:** Xiao SUN, Qizhi CHANG, Chang WANG, Wenhao SHAO, Feng FENG

**Affiliations:** 1.中国质量检验检测科学研究院食品安全研究所，北京 100176; 1. Institute of Food Safety，Chinese Academy of Quality and Inspection & Testing，Beijing 100176，China; 2.国家市场监督管理总局重点实验室 （食品质量与安全），北京 100176; 2. Key Laboratory of Food Quality and Safety，State Administration for Market Regulation，Beijing 100176，China; 3.西安市食品药品检验所，陕西 西安 710119; 3. Xi’an Institute for Food and Drug Control，Xi’an 710119，China; 4.中国医科大学药学院，辽宁 沈阳 110122; 4. School of Pharmacy，China Medical University，Shenyang 110122，China

**Keywords:** 高效液相色谱-串联质谱, 电子烟油, 咖啡因, 牛磺酸, high performance liquid chromatography-tandem mass spectrometry （HPLC-MS/MS）, electronic cigarette oil, caffeine, taurine

## Abstract

为保护消费者健康，电子烟油中禁止添加咖啡因和牛磺酸。然而，由于这两个化合物极性差异大，针对它们的同时检测仍面临挑战。本研究通过筛选和优化不同功能团的色谱柱及其分离条件，使用同时具有多种分离模式的色谱柱，建立了高效液相色谱-串联质谱同时检测电子烟油中咖啡因和牛磺酸的分析方法。样品经50%乙腈水溶液提取，以乙腈和0.1%甲酸水溶液（含10 mmol/L甲酸铵）作为流动相梯度洗脱，使用FMD Comixsil ACRP色谱柱（100 mm×2.1 mm，3.0 µm）分离，在多反应监测模式下，通过串联质谱检测器进行检测，外标法定量。结果表明，咖啡因和牛磺酸在色谱柱上都得到了很好的保留，且峰形好，无拖尾。咖啡因在1.00~100 μg/L范围内线性关系良好（*r=*0.997），牛磺酸在10.0~1 000 μg/L范围内线性关系良好（*r*=0.998）。咖啡因、牛磺酸的检出限分别为0.100、1.00 mg/kg，定量限分别为0.250、2.50 mg/kg。在1倍、2倍、10倍3个添加水平下，各组分回收率为88.2%~99.0%，相对标准偏差（RSD）为2.2%~6.6%（*n*=6）。本方法简单、快速，灵敏度高，可为电子烟的质量监管提供技术支撑。

## Abstract (en)

Electronic cigarette refers to electronic delivery systems designed to generate aerosols for human inhalation. In recent years， the global electronic cigarette market has experienced rapid expansion， drawing widespread international attention to its potential health impacts. To protect consumer health， China’s mandatory National Standard for electronic cigarettes issued in 2022 explicitly prohibits the addition of additives and stimulants associated with energy and vitality in electronic cigarette oil. However， to attract consumers， many illicit manufacturers illegally incorporate various stimulating substances into e-liquids. While detection methods have been reported for some illegal additives such as industrial cannabinoids and sweeteners in electronic cigarette oil， there is no fast and reliable detection method for the determination of caffeine and taurine in electronic cigarette oil available to this day. In order to ensure human health， a method was developed for the simultaneous determination of caffeine and taurine in electronic cigarette oil using high performance liquid chromatography-tandem mass spectrometry （HPLC-MS/MS） with a mixed-functional column. Sample preparation involved a simple dilution and filtration step， which was optimized prior to analysis. The analytes were separated on a FMD Comixsil ACRP column （100 mm×2.1 mm， 3.0 μm）. The column temperature was maintained at 30 ℃. The mobile phase consisted of acetonitrile and a 0.1% formic acid solution containing 10 mmol/L ammonium formate with gradient elution. The flow rate was 0.2 mL/min and the injection volume was 1μL. Mass spectrometric detection was carried out using electrospray ionization in positive mode （ESI^+^） with multiple reaction monitoring （MRM）. Under the optimized conditions， both caffeine and taurine were well retained on the chromatographic column， exhibiting excellent， tailing-free peak shapes. The method demonstrated that the limits of detection of caffeine and taurine were 0.100 and 1.00 mg/kg， and the limits of quantification were 0.250 and 2.50 mg/kg， respectively. The linear correlation coefficient （*r*） was ≥0.997. The average recovery of each component was 88.2%-99.0%， with relative standard deviation （RSD， *n*=6） between 2.2% and 6.6%. These results indicate that the method is simple， rapid and accurate. It has been successfully applied to the detection of caffeine and taurine in real electronic cigarette oil samples.

电子烟是指用于产生气溶胶供人抽吸的电子传输系统^［1］^。近年来，全球电子烟市场规模发展迅速，其对人体的健康影响也随之引起世界各国的广泛关注。为保护消费者健康，我国在2022年发布的《电子烟》强制性国家标准中明确规定电子烟油中禁止添加与能量和活力有关的添加剂和兴奋剂^［2］^。然而，为吸引抽吸者购买，许多不法商家会在电子烟油中非法添加具有兴奋作用的各种物质，如咖啡因、牛磺酸、工业大麻等^［3，4］^。针对电子烟油中的工业大麻等非法添加成分，目前已有相关的检测技术报道^［5，6］^，但是对于电子烟油中的咖啡因和牛磺酸等成分，尚缺乏相关的检测技术，这给电子烟的监管带来巨大挑战。

咖啡因和牛磺酸是目前食品中允许添加的成分，其结构式见图1，由于其极性差异较大，所以目前文献中针对咖啡因和牛磺酸的同时检测方法报道较少，大多都是针对咖啡因或牛磺酸分别建立检测方法。这些方法中使用的检测技术主要包括薄层色谱法^［7］^、分光光度法^［8］^、氨基酸分析仪^［9-11］^、毛细管电泳法^［12，13］^、高效液相色谱法^［14-19］^、液相色谱-质谱联用法等^［20-25］^。在这些方法当中，薄层色谱法和分光光度法设备简单，成本低廉，但灵敏度较低；氨基酸分析仪仅能用于牛磺酸的检测；高效液相色谱法应用相对广泛，但由于牛磺酸无紫外吸收或荧光发射，采用液相色谱分析测定时需对牛磺酸进行衍生化处理，衍生操作烦琐。液相色谱-质谱联用法无需衍生化处理，专属性好，灵敏度高，是近些年备受关注的检测方法，但目前已报道的同时测定牛磺酸及咖啡因的分析方法^［24，25］^均使用反相C_18_色谱柱进行分离，牛磺酸在这些色谱柱上没有保留，在实际样品检测时易受样品基质的干扰。本研究针对电子烟这一特殊基质，通过使用一种具有多种分离模式的新型液相色谱柱，建立了能够同时保留和分离牛磺酸和咖啡因的高效液相色谱-串联质谱（HPLC-MS/MS）检测方法，并成功用于市售电子烟油的检测。

**图1:**
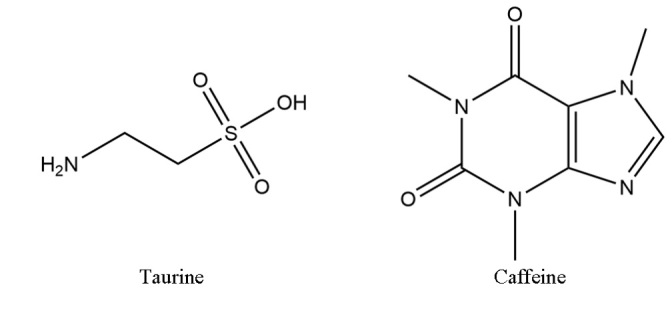
牛磺酸和咖啡因的结构式

## 1 实验部分

### 1.1 仪器、试剂与材料

SCIEX Triple Quad 7500 QTRAP液相色谱-三重四极杆质谱仪（美国SCIEX公司）；分析天平XP 105（瑞士Mettler公司）；Milli-Q Advantage A10超纯水机（美国Millipore 公司）。

咖啡因标准物质（纯度99.7%）购自坛墨质检标准物质中心，牛磺酸标准物质（纯度98.4%）购自天津阿尔塔科技有限公司；甲酸铵、乙腈、甲酸均为色谱纯，购自上海安谱实验科技股份有限公司；实验用水为超纯水；电子烟油样品购自线下实体销售网点。

### 1.2 实验条件

#### 1.2.1 标准储备液与混合标准溶液的配制

分别精密称取咖啡因、牛磺酸标准物质适量，用水溶解并定容至10 mL，配制成质量浓度为1 000 mg/L的标准储备液。-18 ℃避光保存。依次准确吸取上述2种标准储备液适量，用50%乙腈水溶液定容至25 mL，配制成咖啡因1 mg/L、牛磺酸10 mg/L的混合标准溶液。准确吸取上述混合标准物质溶液适量，用50%乙腈水溶液配制成咖啡因质量浓度为1.00~100 μg/L、牛磺酸质量浓度为10.0~1 000 μg/L的系列标准工作溶液，备用。

#### 1.2.2 样品前处理

称取电子烟油样品约0.20 g（精确至0.001 g），加入50 mL 50%乙腈水溶液，涡旋1 min使其混匀后超声提取15 min，经0.22 μm 聚四氟乙烯（PTFE）滤膜过滤，滤液备用。

#### 1.2.3 色谱条件

菲罗门FMD Comixsil ACRP色谱柱（100 mm×2.1 mm，3.0 μm）；流动相：A为乙腈，B为0.1%甲酸水溶液（含10 mmol/L甲酸铵）；梯度洗脱程序：0~2.0 min，95%A；2.0~7.0 min，95%A~70%A；7.0~10.0 min，70%A。柱温：30 ℃；流速：0.2 mL/min；进样量：1 μL。

#### 1.2.4 质谱条件

采用电喷雾离子源正离子模式（ESI^+^），多反应监测（MRM），雾化气、干燥气和加热气均为氮气，气帘气（CUR）压力为276 kPa （40.0 psi），雾化气（GS1）压力为241 kPa （35.0 psi），辅助气（GS2）压力为483 kPa （70.0 psi），电喷雾电压为3.0 kV，去溶剂温度（TEM）为350 ℃。咖啡因、牛磺酸的详细质谱信息见表1。

**表1:** 咖啡因和牛磺酸的最优质谱参数

Compound	Q1 mass	Q3 mass	DP/V	CE/eV
Caffeine	195.2	110.2	10.0	31.0
		138.1^*^		28.0
Taurine	126.1	44.4	10.0	27.0
		108.0^*^		16.0

∗ Quantitative ion； DP： declustering potential； CE： collision energy.

## 2 结果与讨论

### 2.1 提取溶剂的选择

电子烟油的主要成分是丙三醇、1，2-丙二醇和水^［1，2］^，由于丙三醇、1，2-丙二醇等富含羟基，在进行色谱分离时如果不降低这些成分的含量，会对目标物的保留产生较大影响，为此需要在样品处理时使用适当溶剂对样品基质进行稀释处理。鉴于咖啡因和牛磺酸在水中溶解性较好，文献中一般使用水作为提取溶剂进行样品处理^［14-19］^。然而，如前所述，牛磺酸极性很大，使用反相色谱柱进行分离时其在色谱柱上无保留，对于牛磺酸的色谱分离多使用HILIC色谱柱^［21］^，在此情况下，使用纯水作为提取溶剂时，进样液中水的强洗脱能力会对分离产生影响，导致部分咖啡因的峰形变差。实验比较了乙腈、50%乙腈水溶液的提取效果，发现使用50%乙腈水溶液作为提取溶剂，各目标物在不同加标浓度下的回收率均超过90%，且重复性好。由此说明使用50%乙腈水溶液作为提取溶剂时，能够较好地平衡目标物的溶解性和其在色谱柱上保留行为的稳定性。因此，本研究最终选择50%乙腈水溶液作为提取溶剂。

### 2.2 色谱条件优化

如图1所示，牛磺酸属于含硫氨基酸，极性较强，在常规反相C_18_色谱柱基本不保留，故现有文献分析多采用丹磺酰氯进行柱前衍生或者HILIC色谱柱进行分离；咖啡因是含酮基的杂环结构，适合用C_18_色谱柱进行分离。为实现牛磺酸和咖啡因在色谱柱上的同时保留，本研究对比了Waters ACQUITY UPLC HSS T3（100 mm×2.1 mm，1.7 μm）、Waters ACQUITY UPLC BEH HILIC（50 mm×2.1 mm，1.7 μm）以及FMD Comixsil ACRP（100 mm×2.1 mm，3.0 μm） 3种色谱柱的分离效果，每种色谱柱的流动相条件均进行了优化。结果如图2所示，对于反相的C_18_柱来说，尽管本研究使用了能够耐受100%水相的T3色谱柱进行分离，牛磺酸依旧在色谱柱上无保留。对于具有亲水作用的HILIC色谱柱和同时具有多种分离模式的Comixsil ACRP色谱柱都可以实现牛磺酸和咖啡因的较好保留，但进一步比较可以发现咖啡因在Comixsil ACRP色谱柱上的峰形对称、无拖尾，在HILIC色谱柱上则有明显拖尾现象，详见图2，这说明Comixsil ACRP色谱柱可以获得比HILIC色谱柱更好的分离效果。可能的原因如下：不同于BEH HILIC 色谱柱仅通过使用未键合的亚乙基桥杂化颗粒（BEH）作为固定相增大其对强极性化合物的作用力，Comixsil ACRP色谱柱由于其固定相键合有可电离的酸性/碱性基团，能同时提供阴/阳离子排阻、交换、氢键及亲水相互作用色谱等多种作用模式，这使得咖啡因在Comixsil ACRP色谱柱上表现出了比BEH HILIC 色谱柱更好的分离效果。基质加标实验也表明，使用Comixsil ACRP色谱柱可以更好地将基质干扰峰与目标化合物进行分离（见图3），因此，本研究最终确定使用Comixsil ACRP色谱柱进行目标物的分离。

**图2:**
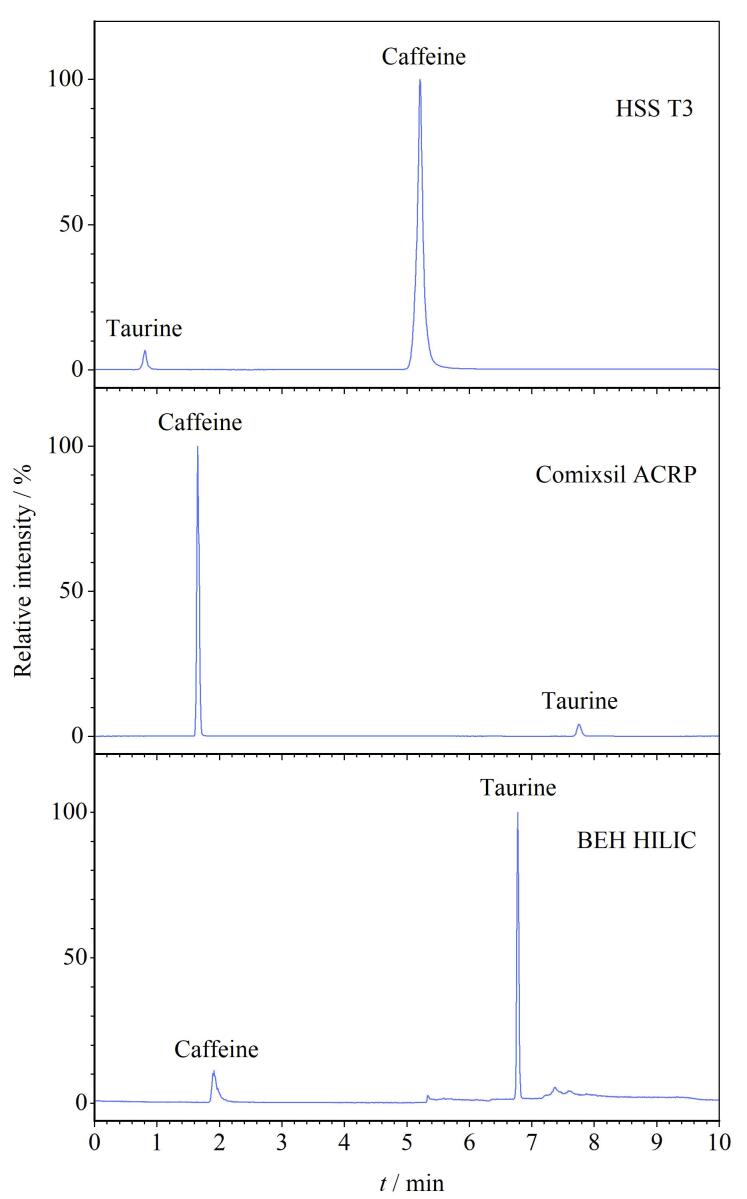
咖啡因和牛磺酸使用3种色谱柱进行分离时的总离子流色谱图

**图3:**
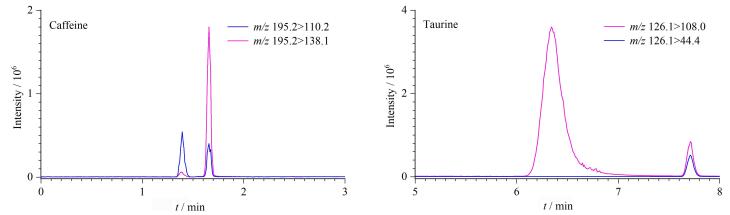
电子烟油空白样品同时加入咖啡因和牛磺酸后的提取离子色谱图

### 2.3 基质效应

基质效应（ME）是质谱检测中普遍存在的现象，其计算公式为ME=（基质匹配混合标准曲线斜率/溶剂标准曲线斜率-1）×100%。若|ME|<20%，表示存在弱基质效应；20%≤|ME|<50%，表示存在中等基质效应；|ME|≥50%，表示存在强基质效应^［20，21］^。本研究取空白电子烟油按照1.2.2小节方法处理样品，得到空白基质溶液，同时用50%乙腈水溶液作为纯溶剂，分别制备标准曲线，根据公式计算基质效应，结果表明，咖啡因的ME为1.4%、牛磺酸的ME为1.8%，均表现出弱基质效应，因此，本方法采用溶剂标准曲线来进行后续的方法学考察。

### 2.4 方法学考察

#### 2.4.1 线性关系、检出限和定量限

将标准工作溶液按照1.2节所述方法测定，以目标组分峰面积为纵坐标（*y*），质量浓度为横坐标（*x*），绘制标准曲线，计算线性回归方程和相关系数。结果显示咖啡因、牛磺酸在各自的线性范围内线性关系良好，相关系数（*r*）分别为0.997、0.998。分别以信噪比（*S/N*）为3和10确定检出限（LOD）和定量限（LOQ），咖啡因、牛磺酸的LOD分别为0.100、1.00 mg/kg；LOQ分别为0.250、2.50 mg/kg。结果见表2。

**表2:** 咖啡因和牛磺酸的线性范围、相关系数、检出限和定量限

Compound	Linear range/（μg/L）	*r*	LOD/（mg/kg）	LOQ/（mg/kg）
Caffeine	1.00-100	0.997	0.100	0.250
Taurine	10.0-1000	0.998	1.00	2.50

#### 2.4.2 回收率和精密度

在空白电子烟油样品中分别添加低、中、高（LOQ、２LOQ、10LOQ）３个水平的混合标准溶液，进行加标回收试验，每个加标水平平行测定６次，并计算回收率和相对标准偏差（RSD），结果见表３。咖啡因的回收率为88.2%~97.2%，相对标准偏差（RSD）≤6.6%；牛磺酸的回收率为95.6%~99.0%，RSD≤3.6%。上述结果表明方法的回收率和重复性良好。

**表3:** 各组分在3水平下的回收率和相对标准偏差（*n*=6）

Compound	Spiked level/（mg/kg）	Recovery/%	RSD/%
Caffeine	0.250	88.2	6.6
	0.500	91.8	6.5
	2.50	97.2	5.3
Taurine	2.50	95.6	3.6
	5.00	95.7	2.2
	25.0	99.0	3.4

### 2.5 实际样品检测

为验证本方法在实际样品检测中的适用性，对随机采购的 10份电子烟油样品进行分析，结果表明所有样品中咖啡因与牛磺酸的响应值均未达到本方法的检出限。这表明当前市场电子烟油产品中咖啡因与牛磺酸的添加风险较低。

## 3 结论

本研究通过比较和筛选出一种同时具有多种作用模式的新型液相色谱柱，建立了电子烟油中咖啡因、牛磺酸的高效液相色谱-串联质谱测定方法，该方法简单、快速，灵敏度和准确度高。方法经实际样品检测验证，具有成本低廉、操作简单、重复性好、准确度高等优点，可应用于电子烟油中咖啡因和牛磺酸的精准检测，对加强电子烟油的质量安全控制具有重要意义。
